# Quality appraisal of clinical practice guidelines for the management of Dysphagia after acute stroke

**DOI:** 10.3389/fneur.2023.1310133

**Published:** 2023-12-05

**Authors:** Shi-Lin Gao, Chang-Qing Liu, Qing-Hua Han, Xiao-Rong Dai, Yi-Wen Liu, Ka Li

**Affiliations:** ^1^West China Hospital, Sichuan University/West China School of Nursing, Sichuan University, Chengdu, China; ^2^Department of Critical Care Medicine, West China Hospital, Sichuan University/West China School of Nursing, Sichuan University, Chengdu, China

**Keywords:** stroke, dysphagia, clinical practice guidelines, AGREE II, quality appraisal

## Abstract

**Objectives:**

Dysphagia is a common complication in stroke patients, widely affecting recovery and quality of life after stroke. The objective of this systematic review is to identify the gaps that between evidence and practice by critically assessing the quality of clinical practice guidelines (CPGs) for management of dysphagia in stroke.

**Methods:**

We systematically searched academic databases and guideline repositories between January 1, 2014, and August 1, 2023. The Appraisal of Guidelines for Research and Evaluation (AGREE II) instrument was used by two authors to independently assess CPG quality.

**Results:**

In a total of 14 CPGs included, we identified that three CPGs obtained a final evaluation of “high quality,” nine CPGs achieved “moderate quality” and two CPGs received “low quality.” The domain of “scope and purpose” achieved the highest mean score (91.1%) and the highest median (IQR) of 91.7% (86.1, 94.4%), while the domain of “applicability” received the lowest mean score (55.8%) and the lowest median (IQR) of 55.4% (43.2, 75.5%).

**Conclusion:**

The CPG development group should pay more attention to improving the methodological quality according to the AGREE II instrument, especially in the domain of “applicability” and “stakeholder involvement;” and each item should be refined as much as possible.

## 1 Introduction

Globally, stroke remained the second-leading cause of death and the third-leading cause of death and disability combined in 2019 (1, 2). Dysphagia is a common complication in stroke patients, widely affecting recovery and quality of life after stroke and increasing mortality risk through increased risk of dehydration, malnutrition and pneumonia (3). The incidence of dysphagia varies widely depending on the method of assessment, compared with clinical assessment (30–55%) and video rheology (64–78%), a lower incidence was detected using initial screening tools (37–43%) (4). However, managing dysphagia correctly and effectively can shorten hospital stays, reduce the risk of death, and decrease healthcare costs (5, 6).

Clinical practice guidelines (CPGs) are a type of declaration that include evidence-informed recommendations aimed at optimizing patient care that are informed by a systematic review of evidence and an assessment of the benefits and harms of alternative care options (4). To date, a number of CPGs have been developed and updated with the aim of ensuring optimal dysphagia management of stroke patients. CPGs would contribute to improving the quality of health care, for example, providing evidence for clinicians to make decisions about patient care and determining appropriate medical criteria, thereby identifying gaps between evidence and practice (7). Nevertheless, hospital personnel adherence to evidence-based stroke care is limited (8), translating evidence into clinical practice is challenging, and implementation of these CPGs in clinical practice remains suboptimal (9, 10).

The quality of the CPGs has a direct impact on utilization (11, 12), and the purpose of this study was to assess the quality of guidelines for managing poststroke dysphagia. Therefore, we used the Appraisal of Guidelines for Research and Evaluation II (AGREE II) instrument (13) to evaluate the quality of CPGs for dysphagia management after stroke, which may be helpful in identifying the potential factors that impact the quality of CPGs. The findings would illustrate the gaps between evidence-based guidelines and clinical practice and attempt to explore potential measures of improvement.

## 2 Materials and methods

### 2.1 Search strategy

A comprehensive literature search was conducted by two authors to identify CPGs for the prevention, diagnosis, and treatment of dysphagia after acute stroke between January 1, 2014, and August 1, 2023. The following databases were searched: PubMed, Web of Science and EMBASE, Clinical Practice Guidelines, the National Institute for Health and Care Excellence, National Guideline Clearinghouse, World Health Organization, Scottish Intercollegiate Guideline Network, New Zealand Guidelines Group and BMJ Best Practice. Search strategies were tailored according to each database (The specific search strategy is displayed in Supplementary File 1). All results were imported into EndNote (Version.X9.2), where duplicates were removed. A third author resolved any disagreements.

### 2.2 Eligibility criteria

The inclusion criteria were as follows: (1) International and national CPGs published on the management of dysphagia after acute stroke; (2) Published or updated from January 1, 2014 to August 1, 2023; (3) Published in English; and (4) Guidelines focused on adult patients. The excluded criteria were as follows: (1) Guideline-related interpretation, application evaluation or brief versions, etc.; (2) Full text not available; and (3) Guidelines under development or withdrawal.

### 2.3 Data screening and extraction

The titles and abstracts of all search results were screened by two authors before checking the full text. In addition, two authors scanned the reference lists of the confirmed papers to identify more relevant CGPs. Then, they extracted the characteristics of the CPGs including year, developer, grading system, country/region, target population, and multidisciplinary team using a predesigned standardized data extraction form.

### 2.4 Quality assessment

The quality of the 14 CPGs was appraised by two authors trained using the AGREE II instrument, which is a reliable tool that is widely used to assess the quality of CPGs (13). AGREE II consists of 23 items organized into six domains and two overall assessment portions. Each item was scored from 1 to 7 (1 = strongly disagree, 7 = strongly agree). Prior to the formal assessment, we discussed the assessment criteria based on the AGREE II manual and training tools to maintain a consistent understanding of each item. After scoring, we organized the CPGs and randomly cross-checked 10% (14) to ensure consistency between authors, especially for items with wide variations in scoring.

The standardized scores for each domain were computed based on the achievement scores (13), as follows: The maximum possible score of domain = 7 (strongly agree) × number of items × number of evaluators; a minimum possible score of domain = 1 (strongly disagree) × the number of items x the number of evaluators. The standardized scores = (obtained score ─ minimum possible score)/(maximum possible score ─ minimum possible score) × 100%.

The AGREE II manual does not offer any advice on how to explain the scores. In accordance with previous studies (15, 16), if a CPG scored above 70% on six domains, it was classified as ‘high quality’; if a CPG scored above 70% on three to five domains, it was classified as ‘moderate quality’; and if a CPG scored less than 70% on ≥ two domains, it was classified as ‘low quality’.

### 2.5 Statistical analysis

All data were analyzed using IBM SPSS Statistics Version 26.0 software and Microsoft Excel 2021. Mean, median and interquartile range (IQR) were computed for the domain scores. The intraclass correlation coefficient (ICC) was computed to measure the interrater agreement when performing a quality appraisal of the CPGs among the two appraisers to ensure the reliability of our conclusions. The level of ICC was classified according to commonly cited cutoffs: poor (< 0.50), fair (0.50–0.75), good (0.75–0.90) or excellent (0.90–1.00) (17).

## 3 Results

**Figure 1:**
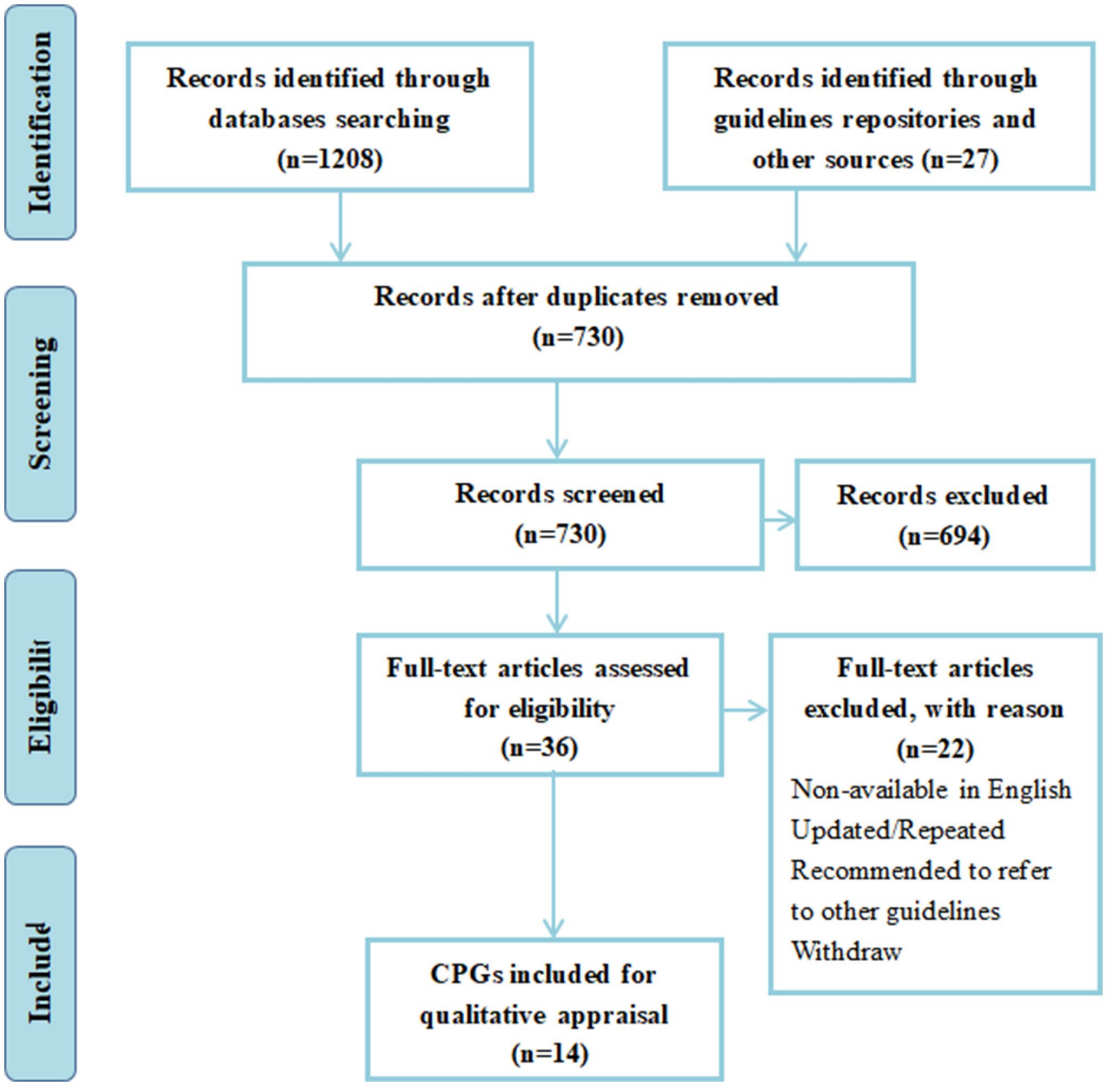
PRISMA flow chart of searching and selecting guidelines.

A total of 1,208 titles and abstracts were generated through database and manual searches. After deleting duplicates, 730 articles were filtered by title and abstract. A total of 36 full-text CPGs were screened for eligibility, and 14 CPGs were included in our systematic review. Figure 1 provides the PRISMA flow chart (18). Table 1 shows the general characteristics of the CPGs included in the analysis. Regarding geographical distribution, six of them are from Europe, the US and Canada all have two CPGs each, while Brazil, China, Turkey, Australia and New Zealand have only one.

**Table 1:** General characteristics of the CPGs included in the analysis.

No.	Year	Developer	Country/Region	Grading system	Evidence based	Intended population	Multidisciplinary team
1	2022	BAN (19)	Brazilian	Evidence: A, B,C Recomm: Class I-III	Clinical trials, meta-analyses, and systematic reviews	Health professionals	Not reported
2	2020	IMSWT (20)	China	Evidence: 1–4 Recomm: GRADE	Existing guidelines and systematic reviews	Medical practitioners, including Chinese herbal medicine specialists, acupuncturists, integrative medicine practitioners, physicians, physical therapists, and clinical pharmacists	Traditional Chinese medicine, integrative medicine, neurology, neurovascular intervention, neurosurgery, emergency neurology, rehabilitation, acupuncture, nursing, pharmacy, evidence-based medicine, and standardization of Chinese medicine and health economics.
3	2022	PMR (21)	Turkey	Evidence: 0–10 Recomm: OC, AC, OD	A 3-round Delphi questionnaire/survey, expert consensus	Not reported	4 physical medicine and rehabilitation medical doctors, consultant experts
4	2016	NICE (22)	UK	Not reported	Not reported	Healthcare professionals, Commissioners and providers of services, People who have had a stroke, their families and carers	Not reported
5	2021	ESO (23)	Europe	Evidence: high, moderate, low, very low Recomm: Strong/Weak	Systematic reviews, meta-analysis, RCTs	Physicians, speech-and-language therapists as well as stroke-nurses, and all the members of the multidisciplinary team	a phoniatrician, a surgeon, two neurologists, a geriatrician, a gastroenterologist, a stroke physician, a pharmacist and a rehabilitation physician
6	2021	ESPEN (24)	Europe	Evidence: 1–4 Recomm: A, B,0,GPP	Not reported	Hospitals, rehabilitation centers, and nursing homes	Six physicians and five dietitians
7	2018	ESPEN (25)	Europe	Evidence: 1–4 Recomm: A, B,0,GPP	Systematic reviews and meta-analysis	Patients with dysphagia and malnutrition	Clinical nutrition, Neurology, Geriatrics, Dietetics and Intensive Care
8	2017	NSF (26)	AN	Recomm: Weak, Strong	Systematic reviews and RCTs	Healthcare professionals	Clinical expert, people with relevant lived experience
9	2016	RCP (27)	UK	Not reported	All high-quality evidence available	Clinicians, patients and their families and carers, and those with responsibility for commissioning stroke services	Clinicians, people with stroke and their families
10	2016	AHA/ASA (28)	USA	Evidence: A, B, C Recomm: Class I-III	Not reported	The members of the multidisciplinary team	Stroke patient, caregivers, physicians, nurses, occupational therapists, recreation therapists, nutritionists, social workers,
11	2019	AHA/ASA (29)	USA	Evidence: A, B, C Recomm: Class I-III	Existing systematic reviews, meta-analysis and RCTs	Prehospital care providers, physicians, allied health professionals, and hospital administrators	Not reported
12	2020	CSA (30)	Canada	Evidence: A, B, C Recomm: Not reported	Systematic reviews, meta-analyses, RCTs, and observational studies	People who have already had a moderately or severely disabling stroke	Stroke neurologists, a geriatric psychiatrist, a clinical pharmacologist, neuropsychologists, physiotherapists, occupational therapists, a speech-language pathologist, nurses,
13	2022	CSA (31)	Canada	Evidence: high, moderate, low, Recomm: Strong/ Weak	Systematic reviews, meta-analyses, RCTs, and observational studies	All healthcare providers, health system leaders and planners, and people living with stroke	Seven people with stroke and one caregiver
14	2021	GSN (32)	German	Not reported	RCTs, cohort studies, systematic meta-analysis, and guideline publications	Not reported	Dysphagia experts from 27 medical societies

BAN, Brazilian Academy of Neurology; IMSWT, Integrative Medicine for Stroke working team; GPP, good practice points; GRADE, The Grading of Recommendations Assessment, Development and Evaluation; PMR, Turkish Society of Physical Medicine and Rehabilitation; WGO, World Gastroenterology Organization; CPGs, clinical practice guidelines; NICE, National Institute for Health and Care Excellence; ESO, European Stroke Organization; ESPEN, The European Society for Clinical Nutrition and Metabolism; NSF, National Stroke Foundation; RCP, Royal College of Physicians; AHA/ASA, American Heart Association/American Stroke Association; CSA, Canadian Stroke Association; AN, Australian and New Zealand; GSN, German Society of Neurology.

### 3.1 Quality of CPGs according to the AGREE II domains

Table 2 reports the ICC score, overall quality and recommendation comments of all CPGs. In total, 14 CPGs were included, only three CPGs were found to be of high quality with all domains reaching a score higher than 70%, nine CPGs were graded as moderate quality and the remaining two were classified as low quality. The evaluation results of the two appraisers were reliably consistent, with ICCs (95% CI) ranging from 0.75 (0.48, 0.88) to 0.90 (0.82, 0.99).

**Table 2:** Appraisal of Guidelines for Research and Evaluation (AGREE) II version result for clinical practice guidelines.

CPG	Domain1	Domain2	Domain3	Domain4	Domain5	Domain6	ICC (95% CI)	Overall quality	Recomm Comment
BAN2022 (19)	91.7	66.7	80.2	91.7	75.0	83.3	0.86(0.74–0.95)	Moderate	YES*
NICE2022 (22)	100	38.9	52.1	80.0	64.7	62.5	0.82(0.62–0.92)	Low	NO
IMSWT2020 (20)	94.4	91.7	85.4	88.9	48.0	91.7	0.77(0.58–0.85)	Moderate	YES*
ESO2021 (23)	91.7	86.1	75.0	97.2	22.9	100	0.80(0.58–0.91)	Moderate	YES*
ESPEN2018 (25)	91.7	62.4	65.6	91.7	45.8	95.8	0.75(0.48–0.88)	Moderate	YES*
ESPEN2021 (24)	86.1	72.2	67.7	77.8	54.2	87.5	0.90(0.82–0.99)	Moderate	YES*
NSF2017 (26)	86.1	88.9	67.7	91.7	56.5	100	0.79(0.57–0.91)	Moderate	YES*
PMR2022 (21)	80.6	86.1	50.0	83.3	52.1	83.3	0.87(0.64–0.96)	Moderate	YES*
RCP2016 (27)	91.7	88.9	84.4	97.2	77.0	100	0.75(0.49–0.89)	High	YES
AHA/ASA2016 (28)	94.4	87.4	85.4	100	79.2	87.5	0.78(0.54–0.90)	High	YES
AHA/ASA2019 (29)	100	61.1	78.1	77.8	32.9	83.3	0.85(0.53–0.94)	Moderate	YES*
CSA2020 (30)	88.9	66.7	75.0	83.3	70.8	87.5	0.84(0.68–0.95)	Moderate	YES*
CSA2022 (31)	91.7	86.1	76.0	97.2	81.3	100	0.78(0.54–0.90)	High	YES
GSN2021 (32)	86.1	56.8	43.8	63.9	35.4	100	0.80(0.55–0.91)	Low	NO
Mean	91.1	74.3	70.5	87.3	56.8	90.2			
Median (IQR)	91.7(86.1–94.4)	79.2(62.1–87.8)	75.0(62.2–81.3)	90.3(79.5–97.2)	55.4(43.2–75.5)	89.6(83.3–100)			

YES*, recommended with modifications.

**Figure 2:**
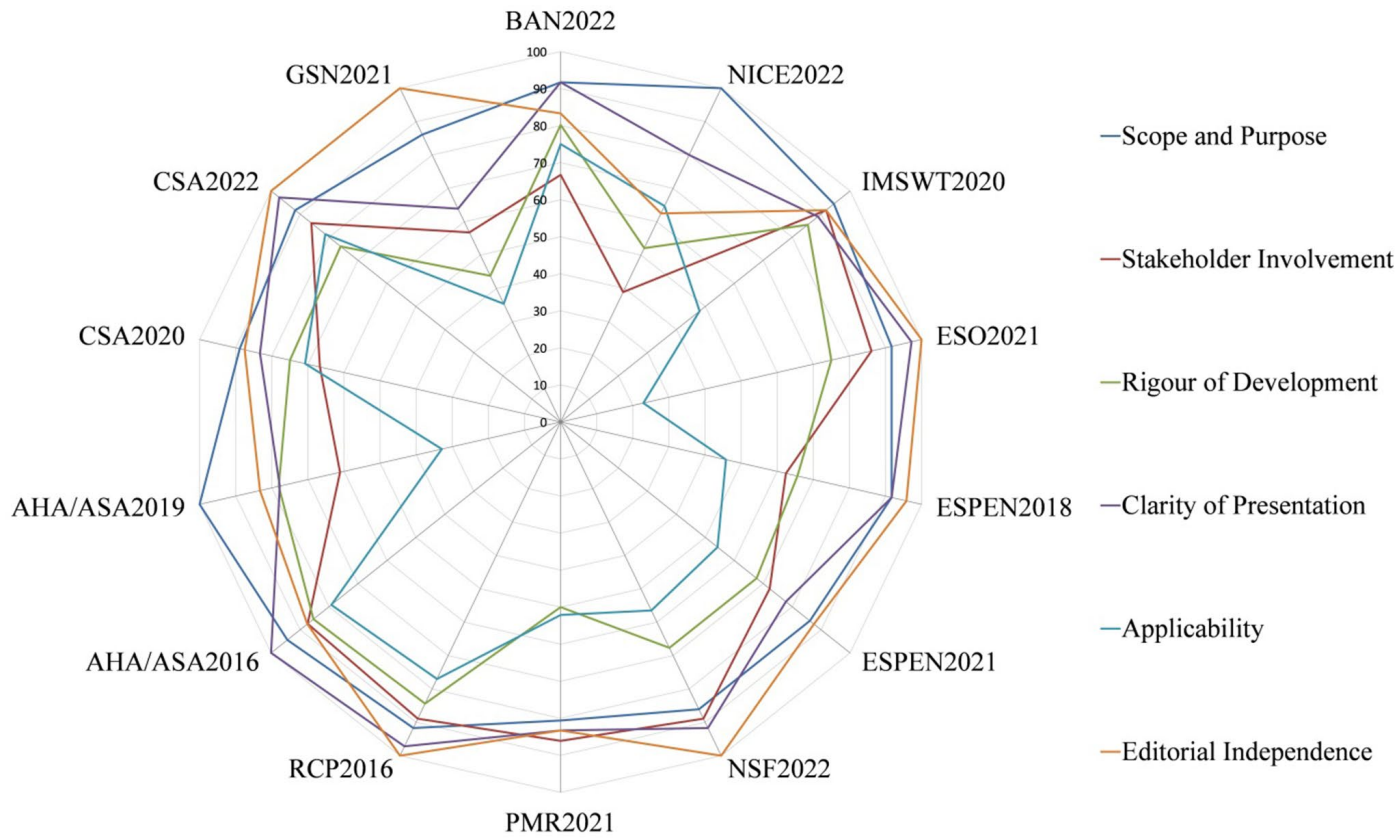
Score distribution of the six domains among the 14 CPGs.

**Figure 3:**
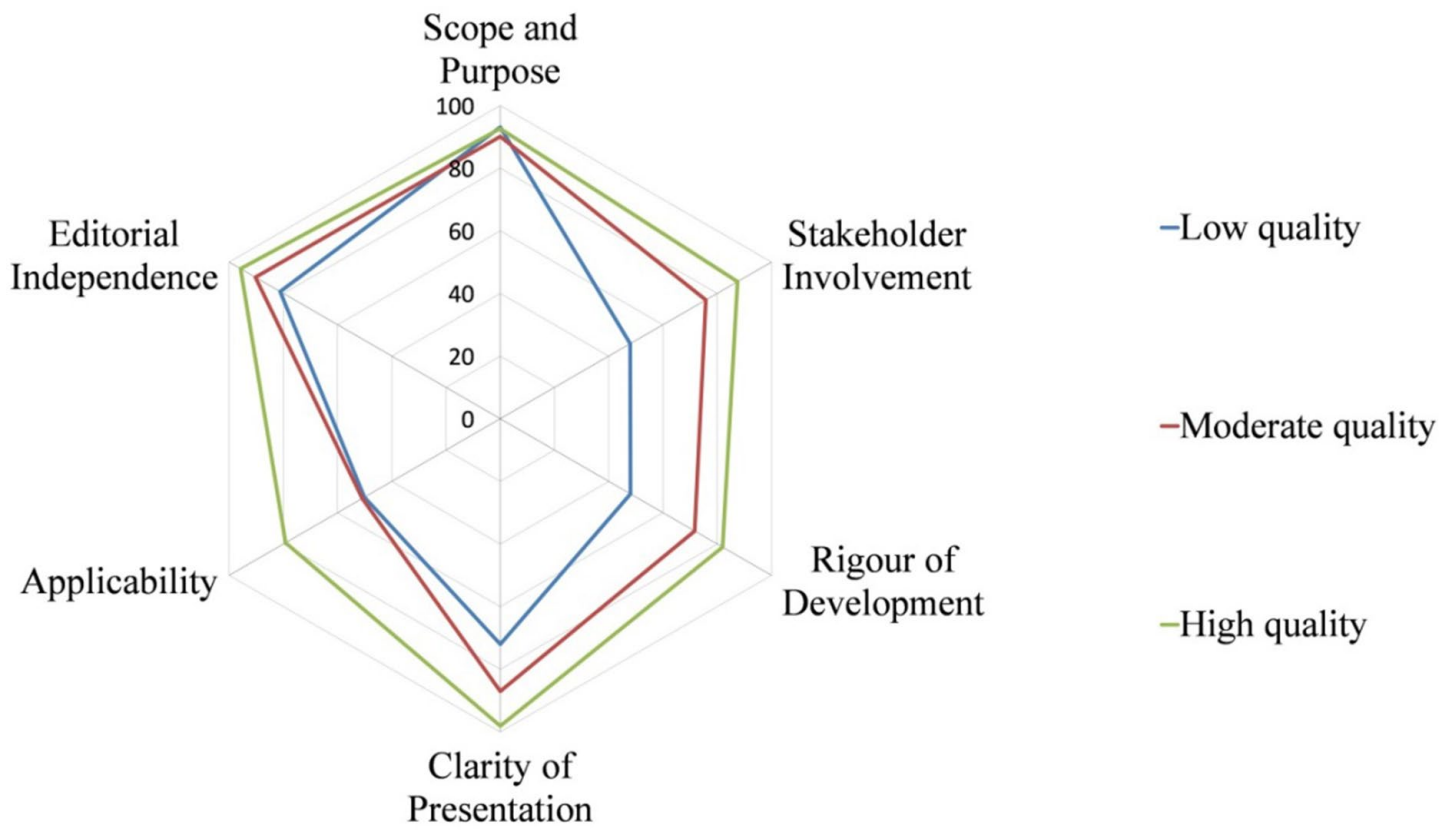
Mean score of each domain for all CPGs sorted by quality classification.

The quality of CPGs evaluated by AGREE II varied widely, not only between guidelines, but also between domains within guidelines. Figure 2 shows the score distribution of the 6 domains among the 14 CPGs. Figure 3 shows the mean score of each domain for all CPGs sorted by quality classification.

#### 3.1.1 Scope and purpose

The domain of “scope and purpose” obtained the highest mean (91.1%) and the highest median (IQR) score of 91.7% (86.1, 94.4%). Moreover, all guidelines achieved over 70% in this domain, but only two of them had a maximum score of 100% (22, 29).

#### 3.1.2 Stakeholder involvement

The standardized scores in this domain ranged from 38.9 to 91.7%, with nine of 14 CPGs scoring above 70%. Most of the poor scores are due to the views and preferences of the target population (patients, public, etc.) have not been sought (22, 32).

#### 3.1.3 Rigor of development

Regarding the standardized scores in this domain, the mean was 70.5%, and the median (IQR) was 75.0% (62.2, 81.3%). GSN2021 (32) obtained the lowest scores (43.8%). Most CPGs lacked clarity in describing all stages of the methodological development or did not provide a procedure for updating the guidelines.

#### 3.1.4 Clarity of presentation

In this domain, the mean score was 87.3%, and the median (IQR) was 90.3% (79.5, 97.2%). Six CPGs received above 90%. In contrast, GSN2021 (32) obtained the lowest score of 63.9%, which means that the guideline development group did not present recommendations clearly.

#### 3.1.5 Applicability

This domain yielded the lowest mean score of 56.8% and the lowest median (IQR) score of 55.4% (43.2, 75.5%). Five CPGs scored more than 70% (19, 27–29, 31), but only CSA2022 (31) scored above 80%, whereas the other guidelines described certain items in the domain unsatisfactorily.

#### 3.1.6 Editorial independence

In this domain, the mean was 90.2%, and the median (IQR) was 89.6% (83.3, 100%). Five CPGs (23, 26, 27, 31, 32) received full marks in this domain, with the exception of NICE2022 (22), which did not explicitly provide information on editorial independence and the competing interests of members of the CPG development group have not been recorded and addressed.

## 4 Discussion

The present study proposes a critical review that evaluates the quality of 14 CPGs developed to manage dysphagia in acute stroke using the AGREE II tool (13). Depending on our results, the quality of CPGs evaluated by AGREE II varied significantly, not only between guidelines, but also between domains within guidelines. RCP2016 (27), AHA/ASA2016 (28) and CSA2022 (31) were classified as high quality and thus were recommended based on the AGREE II tool. Among the domains, “scope and purpose” obtained the highest mean score of 91.1% and the highest median (IQR) score of 91.7% (86.1, 94.4%), while “applicability” yielded the lowest mean score of 56.8% and the lowest median (IQR) score of 55.4% (43.2, 75.5%).

Based on the AGREE II reported items, the domain of “applicability” performed the worst, which is consistent with other quality assessment results of CPGs in different healthcare topics (33, 34). Many CPGs failed to identify and describe the potential facilitators, barriers and advice or tools on how the recommendations can be put into practice. This may be one of the reasons why clinical implementation is not as effective as it could be (9, 35). To address this issue, we find that implementation science approaches are feasible, and a quality improvement intervention that includes online educational videos, mobile health technology, simplified versions of the guidelines manual, audits and feedback, is recommended to improve the CPG adherence of medical staff and patients, user awareness and CPG uptake (36–39).

Regarding the domain of “stakeholder involvement,” some CPGs did not clearly describe the guideline development group or the views and preferences of the target population (patients, public, etc.) were not been sought. During the development of CPGs, patients and a variety of stakeholders, such as clinicians of all types, insurance payers and funders, health policy decision makers, and experts should be involved in the development of CPGs to set priorities, ensure feasibility, and promote distribution and compliance (6, 40, 41).

Most of the CPGs lacked clarity in describing the crucial stages of the methodological development, especially in external review and procedure for updating, which is important for transparency and applicability (42). In addition, guidelines would benefit from a more prescriptive and standardized evidence-based approach to developing recommendations and avoiding the use of ambiguous recommendations. Two of the included CPGs [IMSWT2020 (20), CSA2022 (31)] used the AGREE II tool during the external review and development phase. Although IMSWT2020 (20) used the AGREE II instrument, high quality is still not achieved in the domain of applicability. Therefore, the AGREE II instrument should be considered in the process of planning, developing and publishing CPGs for guideline development groups (13). Our results were largely similar to the results of CPG quality appraisal in different clinical topics (43–45), indicating that the problems in CPG development have some commonality. The CPG development group should pay more attention to improving the methodological quality according to the AGREE II instrument, and each item should be refined as much as possible (16, 42).

In addition to focusing on improving the transparency and methodological rigor of the guideline development process, the quality of guidelines is more dependent on high-quality evidence. However, most of the recommendations in the above guidelines are based on low to moderate quality evidence, and even some of them are not based on evidence. More high-quality evidence is needed for the management of post-stroke dysphagia, such as how to select instruments to evaluate swallowing with sensory tests (29), rational dietary programs (24), and effective therapies (31), which are extremely important for improving the quality of care for patients with post-stroke dysphagia.

Our study has several strengths. First, before the formal assessment, two assessors discussed the appraisal criteria according to the AGREE II manual and training tools to maintain the understanding of each item in line with each other. After scoring, the CPGs were collated with a randomized 10% cross-check (14) to ensure consistency between authors, especially for the items with significantly different scores. Furthermore, to the best of our knowledge, this is the first study that compares and evaluates the quality of CPGs in the nutritional management of stroke patients.

Due to language or publication restrictions, our review is limited to CPGs written in English, and excluding CPGs written in other languages may introduce bias. Furthermore, AGREE II does not provide an explicit cutoff to distinguish between high quality, moderate quality, and low quality CPGs. We defined them based on previous studies, but we are not exempt from misinterpretation that may derive from heterogeneity in the formulation and wording of recommendations. In addition, it is worth noting that in this study, only the critical appraisal of the quality development of the guidelines was performed, without any assessment of the quality of the guidelines’ content.

## Data availability statement

The original contributions presented in the study are included in the article/Supplementary material, further inquiries can be directed to the corresponding author.

## Author contributions

S-LG: Data curation, Methodology, Writing – original draft, Writing – review & editing. C-QL: Methodology, Writing – review & editing. Q-HH: Data curation, Writing – review & editing. X-RD: Data curation, Writing – review & editing. Y-WL: Supervision, Validation, Writing – review & editing, Methodology. KL: Supervision, Validation, Writing – review & editing.
