# Supplementary material for: Quality appraisal of clinical practice guidelines for the management of Dysphagia after acute stroke
Source: Front Neurol. 2023 Dec 5;14:1310133. doi: 10.3389/fneur.2023.1310133 (PMC10728278; doi:10.3389/fneur.2023.1310133)
Supplement: Supplementary file 1 [file Table_1.pdf]

Supplementary File 1. CPGs search strategies for the management of dysphagia after acute stroke.

1. PubMed search strategy

Search	Search terms
#1	"Guideline" [Publication Type] OR "Practice Guideline" [Publication Type]
#2	(guideline*[Title/Abstract]) OR (practice*[Title/Abstract])
#3	#1 OR #2
#4	"Deglutition Disorders"[Mesh]
#5	((((Deglutition Disorders[Title/Abstract]) OR (Dysphagia[Title/Abstract])) OR (difficulty swallowing[Title/Abstract])) OR (swallowing disorder[Title/Abstract])) OR (swallowing dysfunction[Title/Abstract])
#6	#4 OR #5
#7	"Stroke"[Mesh] OR "Ischemic Stroke"[Mesh] OR "Hemorrhagic Stroke"[Mesh]
#8	((((((cerebral stroke[Title/Abstract])) OR (ischemic stroke[Title/Abstract])) OR (cerebral infarction[Title/Abstract])) OR (cerebrovascular disease[Title/Abstract])) OR (vascular accident[Title/Abstract])) OR (hemorrhagic stroke[Title/Abstract])
#9	#7 OR #8
#10	#3 AND #6 AND #9

Results: 239

2. Web of Science

Search	Search terms
#1	MH: (Practice Guidelines) OR MH: (Consensus)
#2	TX: ((practice guideline*) OR (clinical guideline*) OR consensus OR (clinical practice) OR (best practice*) OR (nursing practice))
#3	#1 OR #2
#4	MH: (Deglutition Disorders)
#5	TX: ((((Deglutition Disorders*) OR (Dysphagia*)) OR (difficulty swallowing*)) OR (swallowing disorder*)) OR (swallowing dysfunction*)
#6	#4 OR #5
#7	MH:(Stroke) OR MH:(Ischemic Stroke) OR MH:(Hemorrhagic Stroke)
#8	TX: ((((((cerebral stroke*)) OR (ischemic stroke*)) OR (cerebral infarction*)) OR (cerebrovascular disease*)) OR (vascular accident*)) OR (hemorrhagic stroke*)
#9	#7 OR #8
#10	#3 AND #6 AND #9

Results: 461

3. EMBASE

Search	Search terms
#1	'practice guideline'/exp OR 'consensus'/exp OR 'nursing practice'/exp OR ' clinical practice'/exp
#2	(practice guideline*) OR (clinical guideline*) OR consensus OR (clinical practice) OR (best practice*) OR (nursing practice)
#3	#1 OR #2
#4	' Deglutition Disorders '/exp
#5	(Deglutition Disorders*) OR (Dysphagia*) OR (difficulty swallowing*) OR (swallowing disorder*) OR (swallowing dysfunction*)
#6	#4 OR #5
#7	'Stroke '/exp OR 'Ischemic Stroke'/exp OR 'Hemorrhagic Stroke'/exp
#8	(cerebral stroke*) OR (ischemic stroke*) OR (cerebral infarction*) OR (cerebrovascular disease*) OR (vascular accident*) OR (hemorrhagic stroke*)
#9	#7 OR #8
#10	#3 AND #6 AND #9

Results: 508

4. Guidelines Repository searches

Repository name	Search term(s)
Clinical Practice Guidelines	Deglutition Disorders/ Dysphagia/ difficulty swallowing/ swallowing disorder/ wallowing dysfunction<br><br>vascular accident/ cerebral infarction/ cerebrovascular disease/ stroke
The National Institute for Health and Care Excellence	
National Guideline Clearinghouse	
World Health Organization	
Scottish Intercollegiate Guideline Network	
New Zealand Guidelines Group	
BMJ Best Practice	

Results: 27
